# Low Nitrogen Fertilization Alter Rhizosphere Microorganism Community and Improve Sweetpotato Yield in a Nitrogen-Deficient Rocky Soil

**DOI:** 10.3389/fmicb.2020.00678

**Published:** 2020-04-15

**Authors:** Yanqiang Ding, Yanling Jin, Kaize He, Zhuolin Yi, Li Tan, Lisha Liu, Mingshuang Tang, Anping Du, Yang Fang, Hai Zhao

**Affiliations:** ^1^CAS Key Laboratory of Environmental and Applied Microbiology, Environmental Microbiology Key Laboratory of Sichuan Province, Chengdu Institute of Biology, Chinese Academy of Sciences, Chengdu, China; ^2^Key Laboratory of Bio-Resource and Eco-Environment of Ministry of Education, College of Life Sciences, Sichuan University, Chengdu, China; ^3^University of Chinese Academy of Sciences, Beijing, China; ^4^Sweetpotato Institute, Nanchong Academy of Agricultural Sciences, Nanchong, China

**Keywords:** sweetpotato [*Ipomoea batatas* (L.) Lam.], urea, plant growth-promoting bacteria, biological nitrogen fixation, phytohormone

## Abstract

Sweetpotato can be cultivated in the reclaimed rocky soil in Sichuan Basin, China, which benefits from the release of mineral nutrients in the rocky soil by microorganisms. Shortage of nitrogen (N) in the rocky soil limits sweetpotato yield, which can be compensated through N fertilization. Whereas high N fertilization inhibits biological N fixation and induces unintended environmental consequences. However, the effect of low N fertilization on microorganism community and sweetpotato yield in the N-deficient rocky soil is still unclear. We added a low level of 1.5 g urea/m^2^ to a rocky soil cultivated with sweetpotato, and measured rocky soil physiological and biochemical properties, rhizosphere microbial diversity, sweetpotato physiological properties and transcriptome. When cultivating sweetpotato in the rocky soil, low N fertilization (1.5 g urea/m^2^) not only improved total N (TN) and available N (AN) in the rocky soil, but also increased available phosphorus (AP), available potassium (AK), and nitrogenase and urease activity. Interestingly, although low N fertilization could reduce bacterial diversity through affecting sweetpotato root exudates and rocky soil properties, the relative abundance of P and K-solubilizing bacteria, N-fixing and urease-producing bacteria increased under low N fertilization, and the relative abundance of plant pathogens decreased. Furthermore, low N fertilization increased the phytohormones, such as zeatin riboside, abscisic acid, and methyl jasmonate contents in sweetpotato root. Those increases were consistent with our transcriptome findings: the inhibition of the lignin synthesis, the promotion of the starch synthesis, and the upregulated expression of *Expansin*, thus resulting in promoting the formation of tuberous roots and further increasing the sweetpotato yield by half, up to 3.3 kg/m^2^. This study indicated that low N fertilization in the N-deficient rocky soil improved this soil quality through affecting microorganism community, and further increased sweetpotato yield under regulation of phytohormones pathway.

## Introduction

Sweetpotato is the 7th most important food crop, with a global annual production of more than 1 × 10^11^ kg (Yang et al., 2017). As a rich source of starch, proteins, vitamins, dietary fiber and mineral elements, sweetpotato plays an important role in ensuring food security, especially in sub-Saharan Africa and Asia (Wu S. et al., 2018). With the growing population, global food security is becoming increasingly serious (Food and Agriculture Organization, 2019). Moreover, the global arable land area per capita has decreased from 5.0 × 10^3^ to 2.0 × 10^3^ m^2^ in the past 70 years (FAO). Therefore, further improving the yield of sweetpotato on the limited available land has become of great significance.

Rocky soil has high potential for agricultural utilization due to rich phosphorus (P), potassium (K), iron and other nutrients (Manning and Theodoro, 2018). For example, in the Sichuan Basin, the largest sweetpotato producing area in China (approximately 1.0 × 10^10^ m^2^), more than 70% of the hills (approximately 5.0 × 10^10^ m^2^) are covered by rocky soil, which can be reclaimed for sweetpotato cultivation by local farmers (Deng et al., 2014). However, most of the P and K are fixed in the rocky soil, keeping them from being taken up readily by plants (Chatterjee et al., 2014). More seriously, both total nitrogen (TN) and available nitrogen (AN) are inadequate in the rocky soil due to the lack of nitrogen (N) fertilization and other exogenous N input (Zhu et al., 2008).

Microorganisms play a key role in N fixation, elemental transformation, and improving mineral fertility in soil (Pii et al., 2015; Ciccazzo et al., 2016). Biological N fixation is the second largest N source after N fertilizer in farmland, and approximately 24% total N of crops is derived from the non-symbiotic N fixation of microorganisms (Ladha et al., 2016; Feng et al., 2018). Meanwhile, P and K-solubilizing microorganisms transform unavailable P and K to available P and K (AP, AK) through dissolution and/or mineralization (Etesami et al., 2017; Granada et al., 2018). In addition, microorganisms affect soil pH, density and porosity through metabolic activities (Helliwell et al., 2014). However, most microorganisms are in dormant state in natural soils, especially in the low-organic-matter soil such as rocky soil (Joergensen and Wichern, 2018).

As a barren-tolerate crop, sweetpotato can grow in the rocky soil, and provide organic matter for microorganisms. However, the further improvement of sweetpotato yield may be limited by N deficiency in the rocky soil, especially in the early stage of sweetpotato growth (Taranet et al., 2017), even if AP and AK deficiency could be alleviated by P and K-solubilizing microorganisms (Etesami et al., 2017; Granada et al., 2018). N deficiency can be compensated through N fertilization, but high N fertilization inhibits biological N fixation and induces unintended environmental consequences (Wang et al., 2018; Wu H. J. et al., 2018). Thus, it is reasonably to hypothesize that low N fertilization may increase sweetpotato yield, whereas reducing the impact on environment. However, few investigations have been reported regarding N fertilization of sweetpotato in the N-deficient rocky soil, as well as its effect on microorganism community and sweetpotato yield, and their underlying mechanism remains unknown.

In this study, we added a low level of 1.5 g urea/m^2^ to the N-deficient rocky soil and investigated rocky soil properties, rhizosphere microorganisms, sweetpotato physiological properties and global transcriptional response. The objectives were to (1) investigate the effect of low N fertilization on the bacterial and fungal community in the N-deficient rocky soil, and (2) explore the underlying mechanism by which low N fertilization affects sweetpotato yield in the N-deficient rocky soil.

## Materials and Methods

### Experimental Design

The experiments were conducted in the N-deficient rocky soil at the Yingxi experimental base of the Nanchong Academy of Agricultural Sciences, Sichuan, China (30°52′N, 106°02′E). The rocky soil is a kind of weathered shale with a diameter less than 1.0 cm, and it is easy to be crushed and ploughed (Supplementary Figure S1A). The soil type is purplish soils according to Classification and codes for Chinese soil (GB/T 17296-2009). The rocky soil had a pH of 7.9, total carbon (TC) content of 11.2 g/kg dry soil, dissolved organic carbon (DOC) of 89.2 mg/kg dry soil, TN content of 0.8 g/kg dry soil, AN content of 7.5 mg/kg dry soil, total P (TP) content of 0.6 g/kg dry soil, AP content of 2.5 mg/kg dry soil, total K (TK) content of 17.5 g/kg dry soil, and AK content of 163.6 g/kg dry soil.

Sweetpotato [*Ipomoea batatas* (L.) Lam.] cultivar Nanshu 88 was planted by ridging at a height of 40 cm in a 200 m^2^ field (Luo et al., 2017). The planting density was 6 plants/m^2^. Sweetpotato was planted on June 12, 2016 and harvested on October 20, 2016. The growth period was 130 days. Based on previous experience, a low level of 1.5 g urea/m^2^ was added 35 days after planting (DAP) in the treatment group (BF group), while no urea was added in the control group (CK group). Surface irrigation was applied only when the sweetpotato seedlings were first planted. The local weather conditions during the experiment were shown in Supplementary Table S1.

### Sampling

Sweetpotato was sampled 60 (corresponding to the expansion of tuberous roots) and 130 DAP (corresponding to harvest). Three scattered plots were randomly selected as three replicates. Five sweetpotatoes were excavated from each sample plot. The vines and tuberous roots of sweetpotato were cut and packed separately.

Rocky soil was sampled 0, 60, and 130 DAP. After a sweetpotato was carefully excavated, the shaken-off soil that was not tightly attached to the sweetpotato root was sampled as bulk soil, while the remaining soil that was attached to the sweetpotato root was sampled as rhizosphere soil (Chaudhary et al., 2015). Root fragments and defoliation were carefully removed.

Thus, each sweetpotato and rocky soil sample had three replicates. The tuberous root and rhizosphere rocky soil samples were snap-frozen immediately in liquid N in the field and were then kept on dry ice. All samples were transferred to the laboratory within 3 h and stored in a −80°C freezer for later analyses.

### Chemistry Determination of Sweetpotato

The moisture of sweetpotato tuberous roots and vines was determined by drying at 105°C for 48 h to a constant weight. The sweetpotato root exudates were collected following the method of a previous study (Xu Z. et al., 2015). The carbon (C) and N in root exudates, and TN of sweetpotato tuberous roots and vines was determined by using an elemental analyzer (vario MACRO cube, Elementar, Hanau, Germany). The starch contents of sweetpotato tuberous roots were determined by using a high-performance liquid chromatographer (Thermo 2795, Thermo Corp, Waltham, MA, United States)-evaporative light scattering detector (Alltech ELSD 2000, Alltech Corp, Nicholasville, KY, United States) (Liu et al., 2015). The zeatin riboside (ZR), abscisic acid (ABA), and methyl jasmonate (MeJA) of sweetpotato tuberous roots were extracted and quantified by using an indirect enzyme-linked immunosorbent assay technique (Wang et al., 2012).

The equations for the harvest index and N production efficiency were as follows:

$$\begin{array}{c} \mathrm{Harvest index}\;(\%)=\mathrm{Dry yield of the tuberous root/}\\ \;\mathrm{Dry weight of the whole plant}\times 100; \end{array}$$

N production efficiency (kg/kg) = Fresh yield of the tuberous root/N absorption of the whole plant (Chen et al., 2017).

### Determination of Rocky Soil Properties

The moisture of the rocky soil was determined by drying at 105°C for 48 h to a constant weight. DOC of rocky soil was extracted with distilled water (1:10 w/v, soil/water) and analyzed by a Vario TOC analyzer (Vario MACRO cube, Elementar, Germany) (Xu Z. W. et al., 2015). Bulk soil samples were air-dried for 10 days at room temperature to a constant weight, and sieved (<2 mm) before analyses of soil pH, TC, TN, AN, AP, AK. Rocky soil pH (soil:water, 1:2.5) was determined by using a pH meter (PHS-3C, Fangzhou, China) (Dick et al., 2000). The TC and TN of the rocky soil was determined by using an elemental analyzer (vario MACRO cube, Elementar) (Zhang et al., 2017). The AN of the rocky soil was determined with the micro-Kjeldahl distillation method after digestion with KMNO_4_ and NaOH solution (Subbiah and Asija, 1956). AP and AK were extracted with 0.5 M sodium bicarbonate and 1 M ammonium acetate, respectively, and then measured with inductively coupled plasma optical emission spectrometry (Optima 8300, PerkinElmer, United States) (He et al., 2016). The bulk density of the rocky soil was determined by using micro-computed tomography (micro-CT) scanning (vivaCT80, Scanco Medical, Bruettisellen, Switzerland), and the obtained radiographic images were reconstructed and analyzed by using Scanco software. Nitrogenase activity was detected with a nitrogenase enzyme-linked immunosorbent assay kit (LB5377B, Liborui, China). Urease activity was assayed following the method of a previous study (Zhang et al., 2011).

### Sweetpotato Transcriptome Analysis

The total RNA of sweetpotato root tissue was extracted using TrizolH reagent (Invitrogen, Carlsbad, CA, United States) and then treated with DNase I (Fermentas, Glen Burnie, MD, United States). The RNA concentration, OD260/280 and OD260/230 were measured by using an Agilent 2100 Bioanalyzer (Agilent Technologies, Santa Clara, CA, United States). Qualified libraries were applied for paired-end sequencing (2 × 150 bp) with an Illumina HiSeq 2500 (Illumina, San Diego, CA, United States).

All raw sequencing reads were evaluated with FastQC v0.11.3. The reads containing adapters and low-quality bases were removed. The ribosomal RNA (rRNA) reads were removed by alignment to the rRNA database with Bowtie2 (Langmead and Salzberg, 2012). Approximately 8 Gb of high-quality (Q30 > 92%) clean reads was obtained for each sample after filtering. TopHat2 (version 2.0.3.12) was used to map the clean reads of each sample to the reference genome of *Ipomoea batatas* (GenBank assembly accession: GCA_002525835.2) (Kim et al., 2013; Yang et al., 2017). The fragments per kilobase of transcript per million mapped reads (FPKM) values were calculated by using Cufflinks (version 2.2.1) (Trapnell et al., 2009).

### Microbial 16S and ITS rRNA Sequencing

A PowerSoil DNA Isolation Kit (MOBIO, San Diego, CA, United States) was used to extract total microbial DNA from the rhizosphere rocky soil. The 16S rRNA gene V3−V4 region and ITS2 region were amplified with specific primers (Supplementary Table S2). Qualified libraries were applied for paired-end sequencing (2 × 250 bp) with an Illumina HiSeq 2500 (Illumina).

All raw sequencing reads were evaluated with FastQC v0.11.3. The reads less than 200 bp in length and low-quality reads with a mean quality score below 20 were removed. After filtering, the high-quality reads were assembled into tags (>10 bp overlap and <2% mismatches), and then the tags were filtered using QIIME V1.9.1 (Caporaso et al., 2010). The number of operational taxonomic units (OTUs) was calculated at a distance of 0.03 (97% similarity) by using UPARSE v9.2.64 (Edgar, 2013). The bacterial OTUs were annotated using RDP Classifier v2.2 based on the SILVA database (v128) (Quast et al., 2013) and Greengenes database (July 2010) (DeSantis et al., 2006). Each sample was randomly resampled to 30,000 tags to fairly compare all samples at the same sequencing depth. The fungal OTUs were annotated using RDP Classifier v2.2 based on the UNITE database (version 2016_11_20_ver7) (Abarenkov et al., 2010). Each sample was randomly resampled to 5,000 tags. Alpha diversity indexes (Chao1 index, Sobs, and Shannon index) were calculated using QIIME V1.9.1 (Caporaso et al., 2010).

Tax4Fun was used to predict the bacterial metagenome content based on bacterial composition (Aßhauer et al., 2015), which was calculated with the 16S rRNA gene and annotated with the Greengenes database. The functions were assigned based on the Kyoto Encyclopedia of Gene and Genomes database. FUNGuild was used to parse the ecological guild of the fungal OTUs (Nguyen et al., 2016).

### Quantitative Real-Time PCR

Estimation of *nifH* copy number was performed using quantitative real-time polymerase chain reaction (q-PCR) conducted with a real-time PCR detection system (Bio-Rad Opticon 2, Hercules, CA, United States). The primer pair PolF and PolR was used for q-PCR (Poly et al., 2001). The q-PCR mixtures comprised 12.5 μL of SsoFast EvaGreen Supermix (Bio-Rad), 1 μL each of the forward and reverse primers, 2 μL of template DNA, and 8.5 μL of sterile distilled water for a final volume of 25 μL. The q-PCR conditions comprised 98 °C for 30 s; 40 cycles of 5 s at 95°C, 5 s at 55°C, and 5 s at 72°C; and a final step from 65 to 95°C with an increase of 0.5°C/s to obtain a melting curve. The standard curve was generated by using 10-fold serially diluted plasmids with the *nifH* gene, and the *R*^2^ was 0.996.

### Statistical Analyses

The significant differences in sweetpotato and rocky soil properties, *nifH* copy number and alpha diversity metrics between samples were evaluated using Tukey’s honest significant difference (HSD) test or Student’s two-sample *t*-test in R 3.5.0. Venn diagram and heatmap analysis were performed in R 3.5.0. Spearman’s correlation coefficients between bacterial communities and rocky soil properties were calculated in R 3.5.0, and network analysis was performed with Cytoscape 3.3.0 (Su et al., 2014).

## Results

### Rocky Soil Properties

**FIGURE 1:**
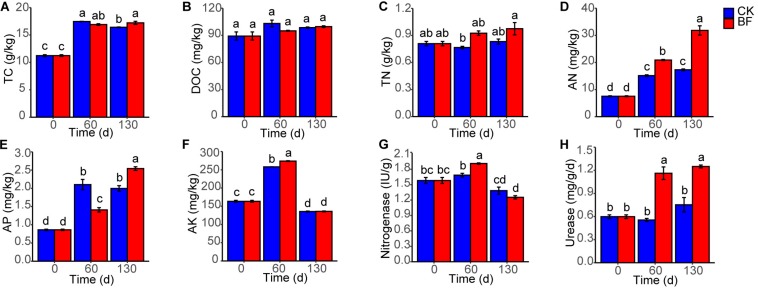
Changes in soil properties. **(A)** TC, total carbon; **(B)** DOC, dissolved organic carbon; **(C)** TN, total nitrogen; **(D)** AN, available nitrogen; **(E)** AP, available phosphorus; **(F)** AK, available potassium; **(G)** nitrogenase; **(H)** urease. Different letters denote significant difference from a Tukey’s HSD test (*P* < 0.05).

The rocky soil properties were improved after low N fertilization (1.5 g urea/m^2^). The TC of BF and CK rocky soil increased from 11.2 to 17.2 and 16.4 g/kg, respectively, and the TC of BF rocky soil was higher significantly than that of CK rocky soil 130 DAP (Figure 1A). The DOC of BF and CK rocky soil also increased from 89.2 to 99.6 and 98.6 mg/kg, respectively (Figure 1B). The TN of BF rocky soil increased from 0.8 to 1.0 g/kg, while that of CK rocky soil changed slightly, and TN of BF rocky soil was always higher than that of CK rocky soil (Figure 1C). The AN of BF rocky soil increased significantly from 7.5 to 31.8 mg/kg, and that of CK rocky soil also increased significantly, but the AN of BF rocky soil was always significantly higher than that of CK rocky soil (Figure 1D). The AP of BF and CK rocky soil increased from 0.9 to 2.5 and 2.0 mg/kg respectively, and the AP of BF rocky soil was lower significantly than that of CK rocky soil 60 DAP, but was higher significantly than that of CK rocky soil 130 DAP (Figure 1E). The AK of BF and CK rocky soil increased from 163.6 to 273.9 and 258.2 mg/kg 60 DAP respectively, then decreased 130 DAP, and the AK of BF rocky soil was higher significantly than that of CK rocky soil 60 DAP (Figure 1F).

The nitrogenase activity of BF rocky soil increased significantly from 1.6 IU (international unit)/g 0 DAP to 1.9 IU/g 60 DAP and was significantly higher than that of CK rocky soil; then, the activity in both of the groups decreased 130 DAP (Figure 1G). The urease activity of BF rocky soil increased from 0.6 to 1.3 mg NH_3_-N/g/d, while that of CK rocky soil changed slightly, and the urease activity of BF rocky soil was always significantly higher than that of CK rocky soil (Figure 1H).

The bulk density of BF and CK rocky soil decreased from 1,077.5 mg/cm^3^ (0 DAP) to 1,038.9 and 1,034.0 mg/cm^3^ (130 DAP), respectively (Supplementary Figure S1). The moisture of BF rocky soil increased from 5.1 to 8.9%, which was almost the same as that of CK rocky soil. The pH of BF rocky soil decreased from 7.9 (0 DAP) to 7.8 (60 DAP) and to 7.6 (130 DAP), while the pH of CK rocky soil decreased to 7.7 (60 DAP) and to 7.8 (130 DAP).

In summary, low N fertilization (1.5 g urea/m^2^) not only improved TN and AN in the rocky soil, but also increased AP, AK, and nitrogenase and urease activity, indicating the enhanced rocky soil fertility.

### Rhizosphere Microorganisms

**FIGURE 2:**
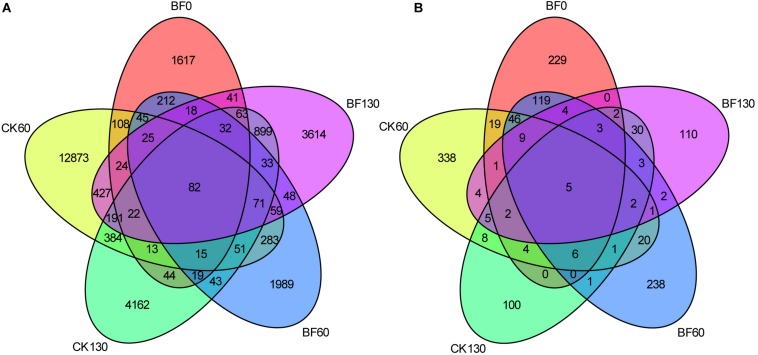
Venn diagram of unique and shared operational taxonomic units (OTUs) among samples. Numbers indicate the number of unique and shared bacterial **(A)** and fungal OTUs **(B)**.

There were abundant bacteria and fungi in the rhizosphere of sweetpotato, and low N fertilization (1.5 g urea/m^2^) affected the diversity of microorganisms, especially bacteria. A total of 27,506 bacterial OTUs were identified, in which, 2,379 for BF0 (CK0), 14,673 for CK60, 3,024 for BF60, 6,123 for CK130, and 5,648 for BF130. The number of shared OTUs between samples was just 82, while most OTUs were unique, especially there were 12,873 bacterial unique OTUs in CK60. These bacterial OTUs belong to 40 phyla and 446 genera (Figure 2A). A total of 1,311 fungal OTUs were identified, in which, 448 for BF0 (CK0), 471 for CK60, 459 for BF60, 171 for CK130, and 182 for BF130. Most fungal OTUs were unique, while just 5 fungal OTUs were shared between samples. These fungal OTUs belong to 6 phyla and 175 genera (Figure 2B).

**FIGURE 3:**
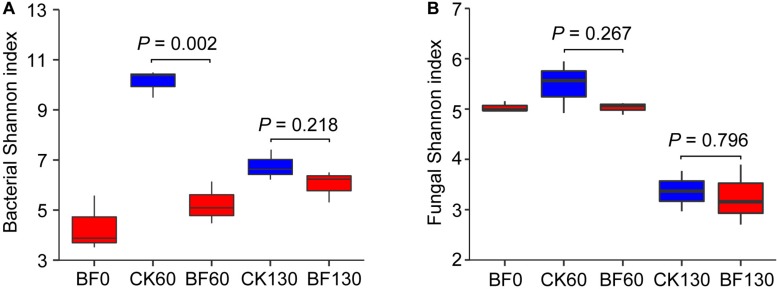
Shannon index of bacteria **(A)** and fungi **(B)**.

Low N fertilization (1.5 g urea/m^2^) reduced rocky soil bacterial alpha diversity in the short term but had little effect in the long term. The bacterial Shannon index of BF rocky soil was 5.8 (60 DAP), which was significantly lower than that (10.7) of CK rocky soil (*P* = 0.002). However, there was no significant difference between the bacterial Shannon indexes of BF and CK rocky soil 130 DAP (6.6 vs 7.4, respectively; *P* = 0.218) (Figure 3A). The changes in the bacterial Chao1 and Sobs metrics were similar to the change in the Shannon index (Supplementary Table S3). However, low N fertilization (1.5 g urea/m^2^) did not reduce rocky soil fungal alpha diversity. There was no significant difference between the fungal Shannon indexes of BF and CK rocky soil 60 DAP (5.0 vs 5.5, respectively; *P* = 0.267) (Figure 3B). The fungal Chao1 and Sobs metrics of BF rocky soil were even higher than those of CK rocky soil 60 DAP (Supplementary Table S3).

**FIGURE 4:**
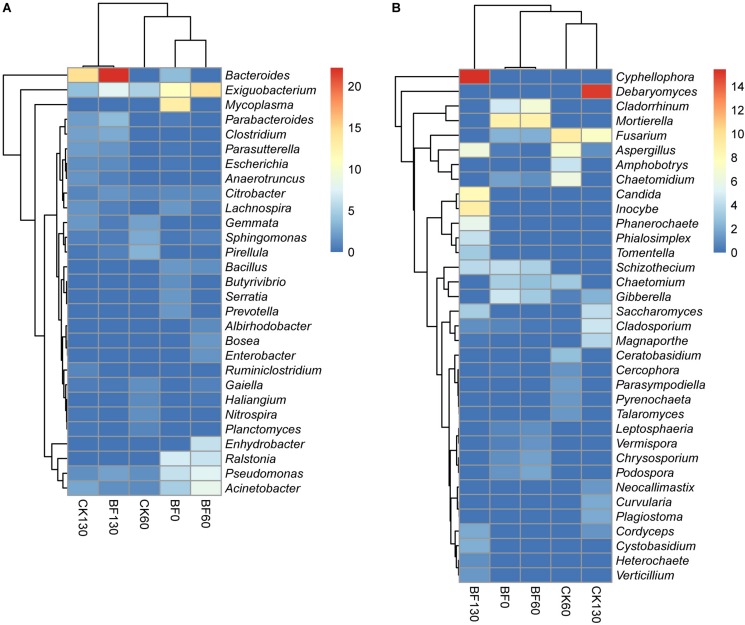
Heatmap analysis of bacterial **(A)** and fungal **(B)** genera.

Sweetpotato cultivation and low N fertilization (1.5 g urea/m^2^) changed the bacterial and fungal community in the rocky soil. For bacteria, the dominant phylum changed from Proteobacteria (37.3% for BF0 (CK0); 62.7% for BF60) to Bacteroidetes (33.8% for BF130). The dominant genus changed from *Mycoplasma* (13.1%) to *Exiguobacterium* (14.4% for BF60) and *Bacteroides* (22.2% for BF130), which were also the dominant genera for CK60 and CK130, respectively (Figure 4A). Heatmap cluster analysis of bacterial genera showed that CK130 and BF130 were grouped together, and BF0 (CK0) and BF60 were grouped together (Figure 4A).

For fungi, the dominant phylum was always Ascomycota during the experiment, while the dominant genus changed from *Mortierella* (9.6% for BF0 (CK0); 10.7% for BF60) to *Cyphellophora* (15.4% for BF130). But the dominant genera of CK60 and CK130 were *Fusarium* and *Debaryomyces*, respectively (Figure 4B). Heatmap cluster analysis of fungal genera showed that BF0 (CK0) and BF60 were grouped together, and CK60 and CK130 were grouped together (Figure 4B).

**FIGURE 5:**
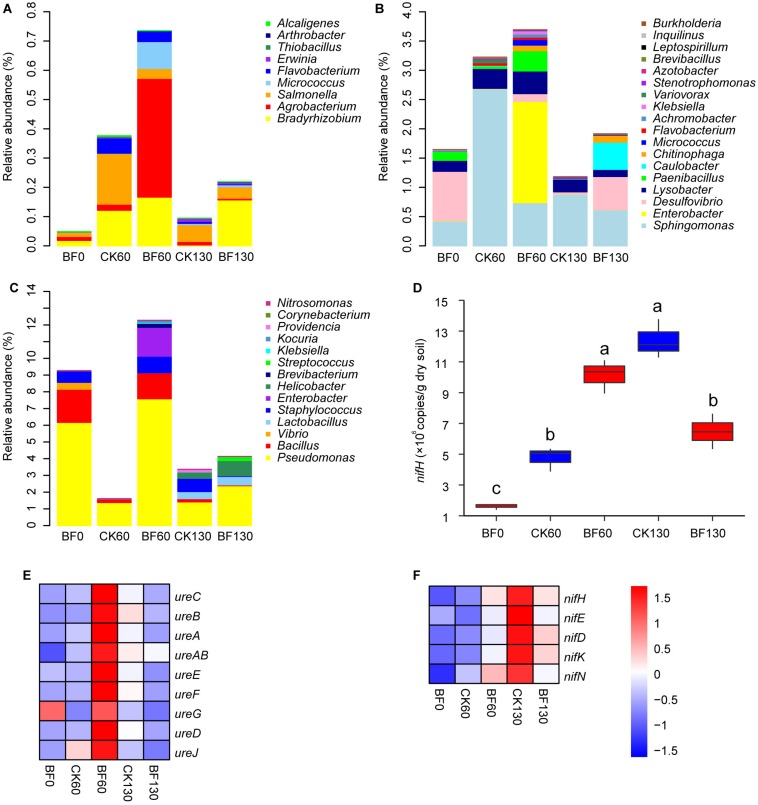
Relative abundance of plant growth-promoting bacteria. **(A)** phosphorus- and potassium-solubilizing bacterial genera; **(B)** N fixing bacterial genera; **(C)** urease-producing bacterial genera; **(D)** *nifH* copy number, different letters denote significant difference from a Tukey’s HSD test (*P* < 0.05); **(E)** relative abundance of urease coding genes; **(F)** relative abundance of nitrogenase coding genes.

More importantly, low N fertilization (1.5 g urea/m^2^) improved the community of plant growth-promoting bacteria. The relative abundances of phosphorus- and potassium-solubilizing bacteria such as *Bradyrhizobium*, *Agrobacterium* and *Salmonella* increased, and the relative abundance of *Bradyrhizobium* in BF rocky soil were higher than these in CK rocky soil 60 and 130 DAP (Figure 5A; Sharma et al., 2013). And the relative abundances of N-fixing bacteria such as *Enterobacter*, *Desulfovibrio* and *Paenibacillus* increased in BF rocky soil and were higher than these in CK rocky soil 60 DAP (Figure 5B). Meanwhile, the relative abundances of urease-producing bacteria in BF rocky soil increased 60 DAP, and were always higher than these in CK rocky soil. Especially 60 DAP, the total relative abundance of urease-producing bacteria in group BF rocky soil was 12.2%, while that in CK rocky soil was only 1.6% (Figure 5C).

The *nifH* copy number was estimated by q-PCR. The *nifH* copy number of BF rocky soil increased significantly from 1.6 × 10^6^ copies/g dry soil 0 DAP to 1.0 × 10^7^ copies/g dry soil 60 DAP, significantly higher than that (4.8 × 10^6^) in CK rocky soil. Then, the copy number decreased to 6.5 × 10^6^ copies/g dry soil 130 DAP, which was lower than that (1.2 × 10^7^) in CK rocky soil (Figure 5D).

The microbiota’s functions were explored by inferring metagenomes with Tax4Fun. The relative abundances of urease (*ureC*, *ureB*, *ureA*, and *ureAB*) and urease accessory protein (*ureE*, *ureF*, *ureG*, *ureD*, and *ureJ*) increased considerably in BF rocky soil and were higher than those of CK rocky soil 60 DAP. The relative abundance of *ureC* in BF 0 DAP was 1.8 × 10^–4^, which increased to 3.5 × 10^–4^ in BF rocky soil 60 DAP, higher than that (2.0 × 10^–4^) in CK rocky soil (Figure 5E). The relative abundance of nitrogenase (*nifH*, *nifE*, *nifD*, *nifK*, and *nifN*) also increased in BF rocky soil and was higher than that in CK rocky soil 60 DAP. While they were lower than those of CK rocky soil 130 DAP. In particular, the relative abundance of *nifH* in BF rocky soil 0 DAP was 2.5 × 10^–5^, and it increased to 5.4 × 10^–5^ in BF rocky soil 60 DAP, higher than that (3.2 × 10^–5^) in CK rocky soil (Figure 5F).

**FIGURE 6:**
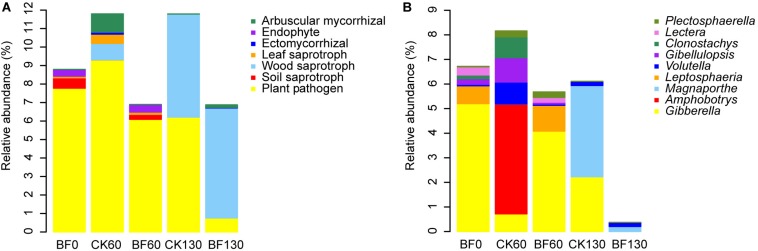
Relative abundance of fungi. **(A)** fungal ecological guild; **(B)** the relative abundance of plant pathogen.

The fungal OTUs were assigned to ecological guild with FUNGuild. The relative abundance of plant pathogen and soil saprotroph decreased, while the relative abundance of wood saprotroph and arbuscular mycorrhizal increased. Especially, the relative abundance of plant pathogen in BF rocky soil decreased from 7.8 to 0.8%, which was lower than that of CK130 (6.2%) (Figure 6A). For example, the relative abundance of *Gibberella* in BF0 (CK0) was 5.2%, which decreased to 2.2% in CK130, however, *Gibberella* was not detected in BF130 (Figure 6B). While the relative abundance of wood saprotroph of BF rocky soil increased from 0.0008 to 5.9% 130 DAP, which was larger than that of CK130 (5.5%) (Figure 6A).

**FIGURE 7:**
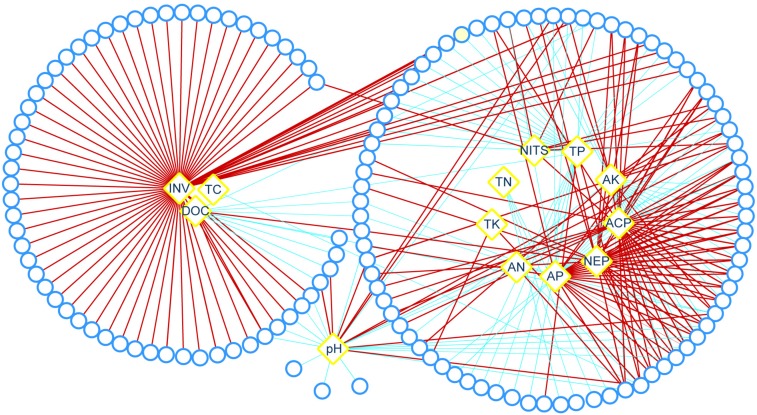
Relationships between bacterial community and soil properties. Red and blue lines represent significant positive and negative correlations, respectively (| Spearman’s ρ| > 0.60; *P* < 0.05), the width of the lines is proportional to the value of Spearman’s ρ, Round represents bacterial genus, rhombus represents soil property. ACP, acid phosphatase; AK, available potassium; AN, available nitrogen; AP, available phosphorus; DOC, dissolved organic carbon; INV, invertase; NEP, neutral phosphatase; NITS, nitrogenase; TC, total carbon; TK, total potassium; TN, total nitrogen; TP, total phosphorus.

Relationships between bacterial community and rocky soil properties were discerned trough network analysis. The result showed that bacteria presented strong relationships with C cycling (invertase, DOC, and TC), mineral element cycling (acid phosphatase, neutral phosphatase, TP, AP, TK, and AK), N cycling (nitrogenase, TN, and AN) and pH (Figure 7). Among those bacterial genera, there were 67 genera presented strong relationships with invertase, and most of which were positive correlations; there were 35, 29, and 29 genera presented strong relationships with AP, acid phosphatase and neutral phosphatase, respectively; and there were 26 genera presented strong relationships with pH (Figure 7).

In summary, low N fertilization (1.5 g urea/m^2^) changed the bacterial and fungal community in the rocky soil, the relative abundance of plant growth-promoting bacteria increased, while that of plant pathogens decreased.

### Sweetpotato Tuberous Root Yield, Physiological Properties and Transcriptional Response

After low N fertilization (1.5 g urea/m^2^), the tuberous root yield of BF sweetpotato increased by half. The tuberous root yields in BF sweetpotato were 0.8 kg/m^2^ and 3.3 kg/m^2^ 60 DAP and 130 DAP, respectively, and the final yield was higher than that (2.2 kg/m^2^) of CK sweetpotato. While the vines yields of BF sweetpotato were 0.5 kg/m^2^ and 1.7 kg/m^2^ 60 DAP and 130 DAP, respectively. And the harvest index (75.9%) of BF sweetpotato was higher than that (70.0%) of CK sweetpotato. The whole-plant N absorption of BF sweetpotato was 8.8 g N/m^2^, which was higher than that (5.9 g N/m^2^) in CK sweetpotato and much higher than the amount of N in the urea applied (0.7 g N/m^2^). The N production efficiency (377.8 kg/kg) of BF sweetpotato was similar to that (387.8 kg/kg) of CK sweetpotato.

**FIGURE 8:**
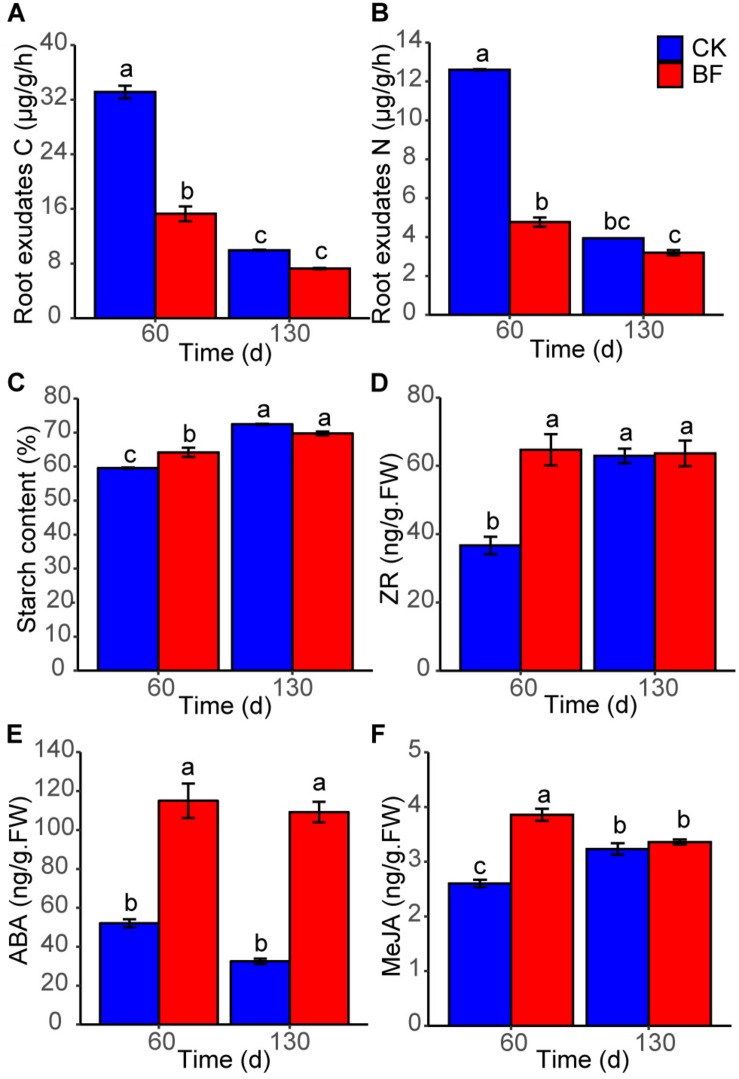
The physiological properties of sweetpotato. **(A)** root exudates carbon; **(B)** root exudates nitrogen; **(C)** starch content; **(D)** ZR, zeatin riboside; **(E)** ABA, abscisic acid; **(F)** MeJA, methyl jasmonate. Different letters denote significant difference from a Tukey’s HSD test (*P* < 0.05).

The root exudates C and N (15.9 and 4.6 μg/g/h) of BF sweetpotato were lower significantly than those (34.0 and 12.6 μg/g/h) of CK sweetpotato 60 DAP, while there was no significant difference 130 DAP (Figures 8A,B). The starch content of BF sweetpotato was significantly higher than that of CK sweetpotato 60 DAP, while there was no significant difference between BF and CK groups when harvesting occurred 130 DAP (Figure 8C).

The phytohormone content of sweetpotato tuberous roots was measured. The ZR content of BF sweetpotato was 64.7 ng/g (fresh weight, FW) 60 DAP, which was higher than that [51.8 ng/g (FW)] of CK sweetpotato (Figure 8D). The ABA contents of BF sweetpotato were always significantly higher than those of CK sweetpotato (BF vs CK: 115.0 vs 52.0 ng/g (FW), 60 DAP; 109.2 vs 32.5 ng/g (FW), 130 DAP) (Figure 8E). The MeJA content [3.9 ng/g (FW)] of BF sweetpotato was significantly higher than that [2.6 ng/g (FW)] of CK sweetpotato 60 DAP, while there was no significant difference between the two groups 130 DAP (Figure 8F).

**FIGURE 9:**
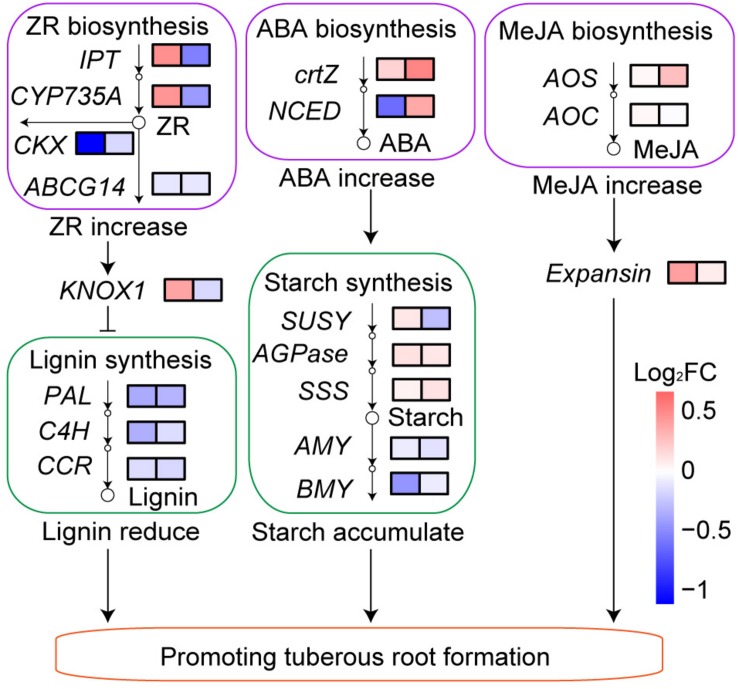
Transcriptional response of sweetpotato to low N fertilization. FC, fold change of the expression level of genes in BF sweetpotato compared with that in CK sweetpotato 60 and 130 DAP.

After low N fertilization (1.5 g urea/m^2^), the expression of ZR biosynthesis genes *IPT* and *CYP735A* was upregulated, while the expression of ZR degradation gene *CKX* and transport gene *ABCG14* was downregulated in BF sweetpotato compared with that in CK sweetpotato (Figure 9). The expression level of *KNOX1* (Homeobox protein knotted-1-like 1) in BF sweetpotato was higher than that in CK sweetpotato 60 DAP, while it was slightly lower 130 DAP. In addition, the expression of lignin biosynthesis genes *PAL*, *C4H*, and *CCR* was downregulated in BF sweetpotato compared with that in CK sweetpotato (Figure 9).

The expression of ABA biosynthesis genes *crtZ* and *NCED* was upregulated in BF sweetpotato compared with that in CK sweetpotato (Figure 9). And the expression of starch biosynthesis genes *SUSY*, *AGPase* and *SSS* was upregulated while the expression of starch degradation genes *AMY* and *BMY* was downregulated in BF sweetpotato compared with that in CK sweetpotato (Figure 9).

The expression of MeJA biosynthesis genes *AOS* and *AOC* was also upregulated in BF sweetpotato compared with that in CK sweetpotato (Figure 9). The expression of *Expansin* was upregulated in BF sweetpotato compared with that in CK sweetpotato (Figure 9).

In summary, low N fertilization (1.5 g urea/m^2^) increased the sweetpotato yield by half, up to 3.3 kg/m^2^. Physiological and transcriptional data indicated that the phytohormones ZR, ABA, and MeJA contents increased in the sweetpotato root.

## Discussion

### Sweetpotato Cultivation and Low Nitrogen Fertilization Alter Microorganism Diversity in the Rocky Soil

Most microorganisms are in dormant state in natural soils, especially in the low-organic-matter soil, and these dormant microorganisms are revived immediately and exhibit increased biodiversity if access to organic matter (Joergensen and Wichern, 2018). Sweetpotato provided organic matter for microorganisms in the form of root exudates (Figures 8A,B), and the soil TC, DOC, TN and AN increased (Figures 1A–D), which might induce the increase in microorganism diversity in the rocky soil. We found abundant bacteria and fungi in the rocky soil, and the number of bacterial OTUs (27,506) was larger than that of fungal OTUs (1,311). And the numbers of shared OTUs between samples were just 82 and 5 for bacteria and fungi, respectively, while most OTUs were unique (Figure 2). These results indicated that the microorganism community varied greatly among different samples, which may be affected by sweetpotato cultivation and low N fertilization (1.5 g urea/m^2^).

Interestingly, low N fertilization (1.5 g urea/m^2^) reduced rocky soil bacterial diversity in the short term but had little effect in the long term, and had a weaker effect on rocky soil fungal diversity (Figure 3 and Supplementary Table S3). Previous studies found that bacteria were primarily influenced by soil properties, while fungi were primarily influenced by plants (Nielsen et al., 2010; Zhalnina et al., 2015). In our study, network analysis showed that bacteria presented strong relationships with rocky soil properties such as pH, AN, AP, and AK (Figure 7), which might affect rocky soil microorganism community. Moreover, as one of the most important source of C and N nutrients for microorganisms (Sasse et al., 2018), the root exudates C and N of BF sweetpotato were just half of those of CK sweetpotato 60 DAP (Figures 8A,B). This result indicated that applying urea inhibited the secreting of sweetpotato root exudates, which might affect rocky soil microorganism community indirectly. However, urea is a quick-acting N source, and the application amount in this study is low, these might explain the little effect on bacterial diversity in the long term (Figure 3A and Supplementary Table S3).

### Microorganism Community Improved Rocky Soil Quality

Microorganisms act as keystone players in improving mineral fertility (such as P and K) and N fixation in soil (Pii et al., 2015; Ciccazzo et al., 2016). The TP and TK in the rocky soil were ample. However, most of the TP and TK combined with minerals and were unavailable for plants (Chatterjee et al., 2014). These unavailable P and K can be transformed to available form by P and K-solubilizing microorganisms through dissolution and/or mineralization (Etesami et al., 2017; Granada et al., 2018). In this study, we found that sweetpotato cultivation and low N fertilization (1.5 g urea/m^2^) improved the relative abundance of P and K-solubilizing bacteria such as *Bradyrhizobium*, *Agrobacterium* and *Salmonella* (Figure 5A) (Sharma et al., 2013). Moreover, AP of BF130 was larger than that of CK130, and AK of BF60 was larger than that of CK60 (Figures 1E,F). Meanwhile, the total relative abundance of P and K-solubilizing microorganisms in BF rocky soil was larger than that in CK rocky soil (Figure 5A). Those results suggested that low N fertilization (1.5 g urea/m^2^) might improve rocky soil AP and AK through P- and K-solubilizing microorganisms.

N fixation by microorganisms is one of the most important source of soil N (Ladha et al., 2016). In our study, the whole-plant N absorption of BF sweetpotato was 3.0 g N/m^2^ higher than that of CK sweetpotato, and the difference was much larger than the amount of N in the urea applied (0.7 g N/m^2^). In addition, both the TN and AN contents in BF rocky soil were higher than those in CK rocky soil (Figures 1C,D). These results indicated that BF rocky soil had higher levels of other sources of N than CK rocky soil, except for the urea applied. This source might be N fixation by microorganisms.

We found that the relative abundances of N-fixing bacteria such as *Enterobacter*, *Desulfovibrio* and *Paenibacillus* in BF rocky soil were higher than these in CK rocky soil 60 DAP (Figure 5B) (Newman and Postgate, 1968; Roesch et al., 2008). And the copy number of *nifH* increased in BF rocky soil and was higher than that in CK rocky soil 60 DAP (Figure 5D). The results from Tax4Fun also supported this finding (Figure 5F). In addition, the nitrogenase activity of BF rocky soil increased and was significantly higher than that of CK rocky soil 60 DAP (Figure 1G). These results suggested that biological N fixation in BF rocky soil was stronger than that in CK rocky soil 60 DAP. Our results were consistent with the results of another study in which urea addition (100 mg/kg soil) increased *nifH* abundance in Antarctic soils, whose TN and AN contents were as low as those in the N-deficient rocky soil in our study (Jung et al., 2011). Our results were also consistent with those from a previous study in which urea fertilization increased *nifH* abundance in a semiarid grassland on the northern Loess Plateau of China, where the TN content was only half of that in the rocky soil that we studied (Zhang et al., 2019). This finding was inconsistent with the findings of previous studies in which N fertilization (30 g N/m^2^/year) inhibited biological N fixation (Wang et al., 2017). The reasons for this difference may be that (1) the original N content in the rocky soil and the level of urea applied to the rocky soil were very low, as in the Antarctic soils and semiarid grassland (Jung et al., 2011; Zhang et al., 2019). A previous study also found that *nifH* gene abundance increased under low N rates (5–15 g N/m^2^/year) but was suppressed under high N rates (50 g N/m^2^/year) (Ning et al., 2015). It was suggested that low N fertilization (0.7 g N/m^2^) may have promoted the growth of N-fixing microorganisms in the N-deficient soil. And (2) the influence on N fixation activity was milder for urea than for nitrate and ammonium (Yamashita et al., 2019).

Urease catalyzes the hydrolysis of urea to ammonia for plant absorption and utilization (Kappaun et al., 2018). Microorganisms are important source of urease in soil (Sun et al., 2019). The relative abundance of urease-producing bacteria in BF rocky soil increased and was greater than that in CK rocky soil (Figure 5C). The Tax4Fun results revealed that the relative abundance of urease and urease accessory protein increased considerably in BF rocky soil after urea was applied (Figure 5E). We also found that the urease activity of BF rocky soil increased to more than 2 times and was always significantly higher than that in CK rocky soil (Figure 1H). These results suggested that applying urea promoted the increase in urease-related microbial relative abundance and further led to the increase in soil urease activity.

Interestingly, low N fertilization (1.5 g urea/m^2^) inhibited plant pathogenic bacteria such as *Mycoplasma* and *Ralstonia* (Figure 4A), which can cause plant yellowing and bacterial wilt, respectively (Whitcomb and Davis, 1970; Genin and Denny, 2012). Moreover, low N fertilization (1.5 g urea/m^2^) inhibited plant pathogenic fungi (Figure 6). For example, *Gibberella* (Figure 6B), which is responsible for many plant diseases causing great economic losses, including bakanae disease of *Oryza sativa* (Nirenberg and O’Donnell, 1998). In addition, sweetpotato cultivation and low N fertilization (1.5 g urea/m^2^) improved the relative abundance of wood saprotroph (Figure 6A), which promoted degradation of organic matter from sweetpotato.

### Increased Phytohormones Promoted the Formation of Sweetpotato Tuberous Roots

Low N fertilization improved soil fertility, especially the AN increased significantly. N is an important nutrient for plant growth, development and production (Leghari et al., 2016). Higher plants have evolved a series of phytohormones such as cytokinins, ABA and MeJA that respond to N signaling and then regulate their physiological and morphological changes (Kiba et al., 2011; Vanstraelen and Benkova, 2012).

Cytokinins are mainly produced in the root and participate in the basic development processes of plants, such as cell division and proliferation, rooting and vascular bundle development (Heyl et al., 2012). N signaling could regulate the growth of each part of the plant through cytokinins (Kiba et al., 2011). In our study, the upregulated expression of ZR (a kind of cytokinins) biosynthesis genes and downregulated expression of ZR degradation and transport genes might led to the higher ZR content of BF sweetpotato than that of CK sweetpotato (Figures 8D, 9). Previous studies found ZR can upregulated the expression level of *KNOX1* (Rupp et al., 1999), and *KNOX1* is known to inhibit lignin synthesis (Townsley et al., 2013). In our study, the expression of *KNOX1* was upregulated, and the expression of lignin biosynthesis genes was downregulated in BF sweetpotato compared with that in CK sweetpotato (Figure 9). Previous study in sweetpotato found that *KNOX1* was upregulated during tuberous root formation, and could promote tuberous root formation (Tanaka et al., 2008). In addition, an important sign of sweetpotato tuberous root formation is the reduction in lignin synthesis (Firon et al., 2013; Ponniah et al., 2017). Thus, ZR might inhibit lignin biosynthesis through *KNOX1* and further promoted the tuberous root formation of BF sweetpotato.

ABA plays a key role in plant stress resistance and the regulation of storage organ and root development, and is closely linked to N signaling (Kiba et al., 2011; Yu et al., 2019). In our study, the upregulated expression of ABA biosynthesis genes might explain why the ABA content of BF sweetpotato was 2–3 times that of CK sweetpotato (Figures 8E, 9). ABA can promote sucrose uptake and unloading in sink organs and promote starch synthesis by increasing the expression of *AGPase* (Akihiro et al., 2005; Nagata and Saitou, 2009). We found the expression of the key enzymes in the starch synthesis pathway, *AGPase* and *SSS*, was upregulated, while the expression of starch degradation genes was downregulated in BF sweetpotato. All of these factors led to starch accumulation in sweetpotato tuberous roots (Figure 9), which further promoted the tuberous root formation of BF sweetpotato.

MeJA is associated with tuberization, fruit ripening and senescence (Wasternack and Hause, 2013). In our study, the upregulated expression of MeJA biosynthesis genes might explain the higher MeJA content of BF sweetpotato 60 DAP (Figures 8F, 9). MeJA was found to promote the expression of *Expansin* in previous research (Han et al., 2012; Ochiai et al., 2013). And *Expansin* causes loosening and extension of plant cell walls, which are instrumental in seed development and yield (Bae et al., 2014). Previous studies also found *Expansin* involve in the initiating tuberous root of sweetpotato (Firon et al., 2013; Ponniah et al., 2017). In our study, with the increase in MeJA content, the expression of *Expansin* was upregulated in BF sweetpotato compared with that in CK sweetpotato (Figure 9), and upregulated expression of *Expansin* promoted the tuberous root formation of BF sweetpotato. All of these factors led to the increase in BF sweetpotato yield.

## Conclusion

In this study, low N fertilization (1.5 g urea/m^2^) in a rocky soil increased the sweetpotato yield by half, up to 3.3 kg/m^2^. We found low N fertilization reduced rocky soil bacterial diversity in the short term through changes of sweetpotato root exudates and rocky soil properties, but had a weaker effect on rocky soil fungal diversity. Moreover, low N fertilization improved rocky soil quality through increasing relative abundance of P and K-solubilizing bacteria, N-fixing and urease-producing bacteria, and decreasing relative abundance of plant pathogens, besides supplying N directly. Furthermore, improved rocky soil quality, especially AN, induced phytohormonal responses of sweetpotato: the ZR, ABA, and MeJA contents in BF sweetpotato root increased, which promoted the formation of tuberous roots and further increased the sweetpotato yield. Our study explored the effect of low N fertilization on improving microorganism community and sweetpotato yield in the N-deficient rocky soil, which could promote sustainable agricultural development and help mitigate the food crisis. Considering the impact of excessive fertilization on the environment, low N fertilization and plant growth-promoting bacteria provide good options for environment-friendly agriculture. Further research should avoid excessive fertilization through searching for plant growth-promoting bacteria and applying fertilizer according to local soil nutrients.

## Data Availability Statement

The raw sequencing data (the transcriptome, 16S and ITS rRNA gene fastq files) are publicly available in the NCBI Sequence Read Archive (SRA) under the SRA accession number SRR10257314–SRR10257319, SRR10257329–SRR10257334, SRR10257376–SRR10257381, SRR9587993–SRR9587998, SRR9588616–SRR9588619, SRR9588634, SRR9588636–SRR9588639, and SRR9588647–SRR9588655.

## Author Contributions

HZ, YF, and KH contributed to the study conception and design. Material preparation, data collection and analysis were performed by YD, YJ, ZY, LT, LL, MT, and AD. The first draft of the manuscript was written by YD and all authors commented on previous versions of the manuscript. All authors read and approved the final manuscript.

## Conflict of Interest

The authors declare that the research was conducted in the absence of any commercial or financial relationships that could be construed as a potential conflict of interest.

## Abbreviations

ABA: abscisic acid
AK: available potassium
AN: available nitrogen
AP: available phosphorus
BF: the treatment group with 1.5 g urea/m^2^
C: carbon
CK: the control group
DAP: days after planting
DOC: dissolved organic carbon
FPKM: fragments per kilobase of transcript per million mapped reads
FW: fresh weight
IU: international unit
MeJA: methyl jasmonate
N: nitrogen
OTUs: operational taxonomic units
q-PCR: quantitative real-time polymerase chain reaction
rRNA: ribosomal RNA
TC: total carbon
TK: total potassium
TN: total nitrogen
TP: total phosphorous
ZR: zeatin riboside.
