# Supplementary material for: Low Nitrogen Fertilization Alter Rhizosphere Microorganism Community and Improve Sweetpotato Yield in a Nitrogen-Deficient Rocky Soil
Source: Front Microbiol. 2020 Apr 15;11:678. doi: 10.3389/fmicb.2020.00678 (PMC7174733; doi:10.3389/fmicb.2020.00678)
Supplement: Supplementary file 1 [file Data_Sheet_1.docx]

Supplementary Material

## Supplementary Figures


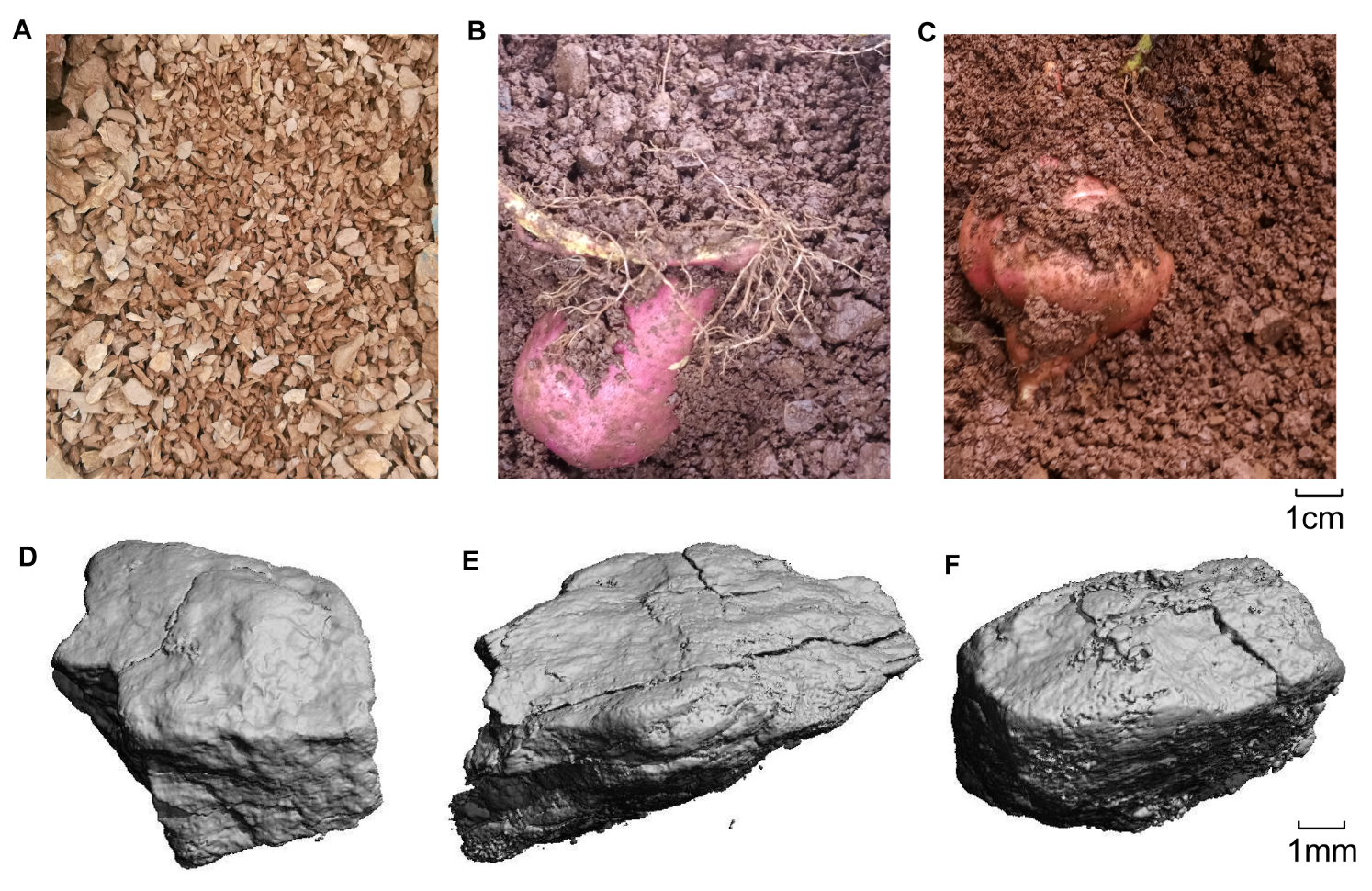


**Supplementary Figure 1.** The rocky soil. **(A)** rocky soil before sweetpotato cultivation (BF0(CK0)); **(B)** rocky soil of CK130; **(C)** rocky soil of BF130; **(D**-**F)**, CT picture of rocky soil of BF0(CK0), CK130 and BF130.

**Supplementary Tables**

**Supplementary Table 1.** Basic meteorological information during the experiment

| Item | Month | | | | |
| --- | --- | --- | --- | --- | --- |
|  | 6 | 7 | 8 | 9 | 10 |
| Mean temperature (℃) | 25.5 | 28.3 | 28.4 | 22.6 | 18.5 |
| Total rainfall (mm) | 104 | 180 | 64 | 75 | 113 |

**Supplementary Table 2.** Primer sequences of 16S, ITS rRNA and *nifH*

|  | Primer sequences |
| --- | --- |
| 16S rRNA | 341F: 5′‐CCTAYGGGRBGCASCAG‐3′ |
|  | 806R: 5′‐GGACTACNNGGGTATCTAAT‐3′ |
| ITS rRNA | F: 5′‐GCATCGATGAAGAACGCAGC‐3′ |
|  | R: 5′‐ATATGTAGGATGAAGAACGYAGYRAA‐3′ |
| *nifH* | PolF: 5’- TGCGAYCCSAARGCBGACTC-3’ |
|  | PolR: 5’- ATSGCCATCATYTCRCCGGA-3’ |

**Supplementary Table 3.** Measures of alpha diversity

|  |  | BF0 | CK60 | CK130 | BF60 | BF130 |
| --- | --- | --- | --- | --- | --- | --- |
| Bacteria | Chao1 index | 3533 | 17453.6 | 8227.8 | 3855.1 | 7142 |
|  | Sobs | 895.3 | 6583.7 | 2298 | 1126.7 | 2127.3 |
| Fungi | Chao1 index | 425.2 | 496.1 | 197.6 | 544.5 | 145.7 |
|  | Sobs | 200 | 206.3 | 98.5 | 223 | 67.7 |
